# Neuroprotective effects of physical activity on the brain: a closer look at trophic factor signaling

**DOI:** 10.3389/fncel.2014.00170

**Published:** 2014-06-20

**Authors:** Cristy Phillips, Mehmet Akif Baktir, Malathi Srivatsan, Ahmad Salehi

**Affiliations:** ^1^Department of Physical Therapy, Arkansas State UniversityJonesboro, AR, USA; ^2^Department of Physiology, Erciyes UniversityKayseri, Turkey; ^3^Department of Psychiatry and Behavioral Sciences, Stanford University School of MedicinePalo Alto, CA, USA; ^4^Department of Biological Sciences, Arkansas State UniversityJonesboro, AR, USA; ^5^VA Palo Alto Health Care SystemPalo Alto, CA, USA

**Keywords:** physical activity, neurotrophins, brain-derived neurotrophic factor, Irisin, FNDC5, myokines, Val66Met

## Abstract

While the relationship between increased physical activity and cognitive ability has been conjectured for centuries, only recently have the mechanisms underlying this relationship began to emerge. Convergent evidence suggests that physical activity offers an affordable and effective method to improve cognitive function in all ages, particularly the elderly who are most vulnerable to neurodegenerative disorders. In addition to improving cardiac and immune function, physical activity alters trophic factor signaling and, in turn, neuronal function and structure in areas critical for cognition. Sustained exercise plays a role in modulating anti-inflammatory effects and may play a role in preserving cognitive function in aging and neuropathological conditions. Moreover, recent evidence suggests that myokines released by exercising muscles affect the expression of brain-derived neurotrophic factor synthesis in the dentate gyrus of the hippocampus, a finding that could lead to the identification of new and therapeutically important mediating factors. Given the growing number of individuals with cognitive impairments worldwide, a better understanding of how these factors contribute to cognition is imperative, and constitutes an important first step toward developing non-pharmacological therapeutic strategies to improve cognition in vulnerable populations.

## Introduction

Man has sought to better understand the relationship between a healthy body and mind for centuries. Exploring this relationship during the pre-Socratic era, the Greek philosopher Thales of Miletus (624–546 B.C.) declared that a happy man is one that possessed a healthy body, a resourceful mind, and a docile nature. Hippocrates (*ca*. 460–370 B.C.) deployed ancient science, philosophy, and craft knowledge to produce the first systematic explanations of human behavior and the body, focusing on both states of health and illness. Later on, Plato (424/423–348/347 B.C.) emphasized the importance of physical exercise for developing the mind. He suggested the harmonious perfection of the body, mind, and psyche could be achieved through physical exercise. Echoing these sentiments, Roman poet Decimus Iunius Iuvenalis (late 1st and early 2nd century A.D.) penned the phrase “Mens sana in corpore sano” and, in so doing, articulated his belief that a sane mind resided within a sound body. Diogenes expanded upon these ideas by emphasizing that diet also contributes to mental and physical prowess. Swiss-German physician Parceleus (1493–1541) advocated for the holistic view treatment of diseases, believing that consideration of the human mind is an integral part of the holistic treatment of disease. As these ideas continued to evolve at the end of the 19th century, the concept of holism began to espouse the notion that stress and mental states have a critical impact upon physical health. Implicit to this conceptualization is the idea that the mind and body are one entity and stressful mental states adversely affect bodily health. Recognizing this, Joseph Pilates (1883–1967), a well-known athlete and author, articulated his belief that achieving a balanced mind and body required more than the absence of disease. Paramount to Pilates conceptualization of wellness was the idea of balance between one’s mind, body, and spirit. Today, modern tools and technologies have been deployed to understand the inextricable relationship between mind and body and, in turn, are elucidating the molecular and cellular mechanisms by which physical exercise alters one important dimension of the mind: cognitive function.

Here we describe current knowledge on the effects of physical activity on cognitive function and the cellular and molecular mechanisms that underlie this relationship. Initially, an overview of the putative mechanisms linking physical activity, cognition, and the subsystems subserving cognitive function prefaces a more intense focus on the neurotrophin signaling hypothesis. This will be followed by a description of studies in both human and animal models that implicate brain-derived neurotrophic factors (BDNF) in these molecular and cellular processes. Finally, cautionary notes regarding the deleterious effects of extreme physical activity are proffered and suggestions for clinical intervention then flank the discussion.

## Putative mechanisms underlying the relationship of physical activity and cognitive function

Numerous studies have reported a robust relationship between high levels of physical activity, hippocampal size, and cognitive measures. Studies in the elderly have shown a direct correlation between increased levels of physical activity and improved cognition, with increases in hippocampal volume following exercise (Erickson and Kramer, 2009), supporting the idea that physical activity leads to anatomical and physiological alterations in the brain. Studies in patients with schizophrenia have demonstrated direct correlations between increased levels of physical activity, increased hippocampal volume, and enhanced spatial memory (Pajonk et al., 2010). Similarly, studies in healthy individuals have demonstrated that high levels of physical exercise are associated with increased hippocampal volume, increased cerebral blood flow, enhanced spatial memory, and reduced brain tissue loss (Colcombe et al., 2003; Pajonk et al., 2010). Although most studies have been performed in adults, a number of studies in school children clearly demonstrate positive correlations between physical activity and academic performance as well (Bass et al., 2013). Thus the benefits of physical activity on cognition appear to be widespread across all age groups. While the dynamic cellular and molecular cascades that underlie the association between physical activity and cognitive function have yet to be fully elucidated, four main hypotheses have been proposed to explain these mechanisms: the cardiovascular, immunologic, neuroendocrine, and neurotrophic signaling hypothesis.

### Cardiovascular effects

The cardiovascular benefits of sustained physical activity include improved exercise capacity, alterations in lipid profiles, reductions in obesity indices, increased rates of heart recovery and variability, reduced resting pulse, and improved blood rheology and hemodynamics (Vuori et al., 2013). Moreover, adequate levels of physical activity are vital for improved small vessel condition (Schmidt et al., 2013), increased cerebral blood flow, and nutrient delivery (Swain et al., 2003). Together, the aforementioned factors are thought to contribute to improved brain health. In support of this idea, Colcombe et al. (2003) reported increased levels of physical activity were inversely correlated with reductions in gray and white matter loss, particularly in the prefrontal, superior parietal, and temporal cortices (Colcombe et al., 2003). Similarly, rodent studies have demonstrated that running exercise lead to improvements in vascular perfusion and angiogenesis in the motor cortex, alterations that were restricted to the motor regions of the cerebral cortex. Other studies have suggested that motor skill training increases synaptogenesis in corresponding regions, while automatic, repetitive motor activities do not (Kleim et al., 1996). For example, motor learning independent of physical activity leads to significant increases in synaptogenesis in the Purkinje neurons in the cerebellum while physical activity independent of motor learning leads to angiogenesis in this region (Black et al., 1990). While cross sectional studies on the relationship between cardiovascular fitness and cognitive function have revealed weak correlations between the two parameters (Hillman et al., 2005), strong and reproducible positive correlations have been consistently revealed in the elderly, suggesting that cardiovascular correlations are stronger when there is a need to restore physical activity to basal threshold levels (Lautenschlager et al., 2008). The idea that the benefits derived from exercise stem from restorative effects of improved cardiovascular function seems plausible given that aged individuals accrue cognitive deficits over time. In aggregate, these findings suggest that physical activity is an important modulator of cognition across the lifespan, but that the contribution of cardiovascular fitness for sustaining healthy cognitive function increases with age by impacting central and peripheral cellular processes.

### Immunological effects

Physical activity results in increased levels of pro-inflammatory, anti-inflammatory cytokines, cytokine inhibitors and chemokines depending upon the intensity and duration of such exercise. The immunological benefits of sustained physical activity include overall enhancement of immune function and anti-inflammatory processes. The importance of the role of exercise in inducing anti-inflammatory effects is underscored by the fact that chronic inflammation has been linked etiologically to cognitive impairment, cardiovascular diseases, and neurodegenerative disorders including Alzheimer’s disease (AD) and Parkinson’s disease (PD; Gleeson et al., 2011). Multiple studies have shown that individuals who regularly participate in physical activity appear to have fewer viral and bacterial infections (DiPenta et al., 2004; Kohut and Senchina, 2004), a lower incidence of systemic low-grade inflammation (Stewart, 2002; Colbert et al., 2004), and a lower incidence of neurodegeneration and cognitive decline (Cotman et al., 2007). Investigating the effects of exercise on immune function, Kohut et al. (2006) studied older adults participating in aerobic or flexibility exercise for 10 months and found a significant reduction in plasma levels of interleukin 6 (IL-6), interleukin 8 (IL-8), C-reactive protein (CRP), and tumor necrosis factor (TNF), linking physical activity to anti-inflammatory processes in the central nervous system (CNS). Similarly, seventeen cross-sectional studies evaluated by Woods et al. (2006) reported an inverse correlation between physical activity and CRP levels. Moreover, brief exercise in young adolescent males has been shown to significantly increase a chemokine protein called chemokine (CXC Motif) ligand 12 (CXCL12) [formerly known as stromal cell-derived factor 1], an important factor involved in angiogenesis (Zaldivar et al., 2007).

While chronic exercise leads to a reduction in chronic inflammation, acute exercise appears to promote a proinflammatory release of cytokines. Performing a meta-analysis, Ploeger et al. (2009) analyzed 19 studies that investigated the effects of acute and chronic exercise on inflammation. They found that individuals diagnosed with type I diabetes mellitus, cystic fibrosis, and chronic obstructive pulmonary disease demonstrated an elevated inflammatory response after participation in a single bout of exercise, whereas patients diagnosed with chronic heart failure and type 2 diabetes mellitus demonstrated an attenuated systemic inflammatory response after participation in chronic endurance training programs. Since the beneficial effects of exercise on cognition usually involves physical activity for a longer duration such as weeks or months, a common mechanism underlying the effects of exercise on cognition might be related to its effects on inflammatory factors IL-6, IL-8, CXCL12, CRP, and TNF.

1) IL-6 is a pro-inflammatory cytokine released in the periphery by T-cells, macrophages, fibroblasts, endothelial cells, and osteoblasts (Burger, 2013). IL-6 plays a critical role in the metabolic regulation of muscle cells and is released in response to eccentric muscle contraction (Febbraio and Pedersen, 2002). Notably, elevation of IL-6 levels has also been shown to be a risk factor for dementia (Ravaglia et al., 2007).

While some studies report that moderate levels of aerobic exercise lead to release of IL-6 from muscle, with circulating levels increasing up to 100-fold for up to 1 h following participation (Pedersen and Fischer, 2007). Yet several other reports indicate such increases of 100 fold occur after a strenuous bout of exercise such as that encountered when running a marathon. Since IL-6 easily crosses the blood brain barrier (BBB; Banks et al., 1994), it could impose significant functional alterations on neurons and glial cells. Rodent studies have shown *IL-6* mRNA and its receptor in pyramidal cells and dentate granule neurons of the hippocampus. IL-6 is expressed constitutively in multiple regions of the CNS that are involved in the mechanisms regulating metabolic, cognitive function, and neuroendocrine changes during physiological conditions (Schobitz et al., 1993). Overexpression of IL-6 in astrocytes in mice led to neurodegenerative alterations and an age-dependent inability to acquire new learning (Heyser et al., 1997). Thus, while strenuous exercise-induced increases in IL-6 could result in inflammation in the brain, a recent report shows that a combination of progressive resistance training and protein enriched diet can reduce circulating levels of IL-6 in the elderly (Daly et al., 2014), suggesting the link between IL-6 levels and exercise requires further investigation.

2) IL-8 or chemokine (CXC Motif) ligand 8 (CXCL8) belongs to CXC family of chemokines and affects many aspects of the immune system, including chemotactic effects on B and T lymphocytes. Additionally, IL-8 appears to promote local angiogenesis in muscle (Akerstrom et al., 2005). Receptors for IL-8 include CXC chemokine receptor 1 (CXCL8R1) and CXC chemokine receptor 2 (CXCL8R2) and are expressed on neurons, glia, and endothelial cells of the BBB. IL-8 enhances neurotransmitter release and inhibits induction of long-term depression in purkinje cells (Giovannelli et al., 1998). Recent work supports the notion that IL-8 might induce neuromodulatory effects. For example, it has been shown that poor memory performance could be linked to reduced serum levels of IL-8 in aged individuals (Baune et al., 2008). Notably, IL-8 increases in response to high-intensity exercise (Ostrowski et al., 2001; Nieman, 2003), but not for moderate intensity exercise (Nieman et al., 2000; Chan et al., 2004).

3) CXCL12 (formerly known as SDF-1) is a pleiotropic chemokine that participates in adaptive immune responses and angiogenesis by recruiting endothelial progenitor cells from the bone marrow (Salcedo and Oppenheim, 2003; Hill et al., 2004). It has been shown that 3 weeks of freewheel training leads to a significant increase in *CXCL12* gene expression, protein levels, and learning and memory in Tg2576 mouse models of AD (Parachikova et al., 2008). CXCL12 is extensively expressed in the CNS, with *CXCL12* mRNA and protein having been detected in cholinergic, dopaminergic, and AVP-ergic neurons in the brain (Callewaere et al., 2008). Accordingly, Tg2576 mice overexpressing mutant *APP* with Swedish mutation show a significant reduction in *CXCL12* mRNA levels in the hippocampus (Parachikova et al., 2008). The question of whether CXCL12 plays a role in the pathophysiology of cognitive dysfunction has been partially addressed by experiments showing that mice chronically treated with CXCL12 antagonist (AMD3100) display significant deficits in learning and memory, as evidenced by their failure in T-maze and novel object recognition (Parachikova et al., 2008).

4) CRP is an acute-phase protein found in the blood, the levels of which play a crucial role in the human immune system. It has been shown that pyramidal neurons of the hippocampus cortical regions express CRP (Yasojima et al., 2000). Interestingly, these neurons show increased expression of CRP in people with AD (Bi et al., 2012). CRP is involved in activation of the complement system and is synthesized primarily in the liver, adipose tissue, and vascular smooth muscle cells (Yasojima et al., 2000). The data on the effects of exercise on CRP are inconsistent. Significant reductions in CRP levels were noted in 11 out of 25 trials of aerobic-based regimens. Furthermore, there were significant CRP reductions associated with physical activity detected in 9 out of 18 adult studies, 4 of 10 child studies, and 1 out of 3 elderly studies (Michigan et al., 2011).

5) TNF (formerly known as tumor necrosis factor-α) is monocyte-derived cytokine that performs a variety of functions in the neuroimmune system, including cell proliferation, differentiation, and cytolysis. It has been shown that elevated levels of TNF are a risk factor for AD in older adults (Tan et al., 2007). Accordingly, a number of studies have linked TNF to AD pathology. It has been shown that reducing the expression of TNF in mouse models of AD leads to a significant reduction in Aβ accumulation (Culpan et al., 2011). Accordingly, a positive correlation has been found between the serum levels of TNF and severity of AD dementia (Guerreiro et al., 2007; Bonotis et al., 2008). Quantification of protein and gene expression for TNF has shown significant increases in the brain cortex in AD (Frankola et al., 2011), yet moderate exercise of 1 month has been shown to reduce levels of TNF (Ambarish et al., 2012). Moreover, moderate exercise in the healthy elderly has been shown to result in a significant reduction in *TNF* gene expression in muscles (Lambert et al., 2008).

Together, the aforementioned studies reveal the presence of a link between exercise and the immune system and implicate this system in many neurobiological processes that underlie cognitive dysfunction, aging, and neurogeneration, extending well beyond classical chemotactic functioning. Among the processes implicated are neuromodulatory and neurotransmitter-like effects along with direct and indirect regulation of neurogenesis.

### Trophic factor signaling and their modulators

#### Neurotrophins

**Figure 1:**
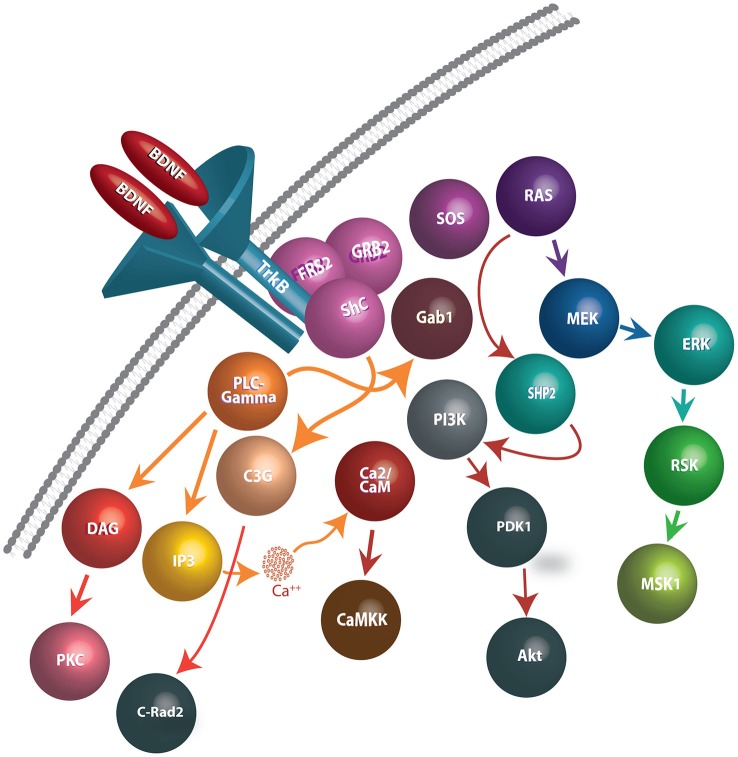
**Schematic representation of BDNF release, binding to TrkB receptors, and downstream events following internalization**. BDNF dimers bind to TrkB receptors and cause autophosphorylation of tyrosine residues on the cytoplasmic domain of TrkB receptors, generating docking sites for several intracellular proteins. In turn, the activation of the TrkB receptors facilitate interactions with Shp2, Shc, and PLC-γmolecules and effectuates the signaling cascades such as PLC/PKC, PI3K/Akt, Ras/Erk, AMPK/ACC and NFB pathways. Signaling pathways involved in BDNF-TrkB interactions include: **(1)** PLC-γ1 signaling: Phosphorylated TrkB receptors bind to PLC-γ1 and lead to its’ activation. PLC-γ1 hydrolyses Phosphatidylinositol (4,5) to generate IP3 and DAG. While IP3 promotes release of Ca^2+^ from internal stores, DAG stimulates DAG-regulated protein kinase C isoforms. **(2)** Ras-MAP/erk signaling: Phosphorylation of Trk receptors provides a recruitment site for binding of the PTB domain of the adaptor protein, Shc. Shc recruits the adaptor protein, Grb2, and complexes with SOS, an exchange factor for Ras (and Rac). Activated Ras stimulates signaling through several downstream pathways, including those mediated by PI3-kinases, Raf, and p38MAP kinase. **(3)** PI-3 kinase signaling: Phosphatidylinositides are generated by PI3-kinase and activate phosphatidylinositide-dependent protein kinase (PDK-1). PDK-1 activates the protein kinase Akt (also known as PKB), which then phosphorylates several proteins important in promoting cell survival.

Improved trophic factor signaling has been considered as the most popular hypothesis to explain the positive effects of physical activity on cognition, with attention centering on the neurotrophins (NTs). NTs are comprised of a closely related family of polypeptides that regulate a variety of neuronal functions including proliferation, survival, migration, and differentiation (Salehi et al., 2003, 2004). The mammalian NTs include nerve growth factor (NGF), BDNF, neurotrophin-3 (NT-3) and, neurotrophin-4/5 (NT4/5). These factors are synthesized by target neurons, initially in the form of pre-pro-protein. The biological actions of NTs are mediated by binding to two different classes of receptor systems, the low affinity P75^NTR^ receptor and the tropomyosin related kinase (Trk) family of high affinity tyrosine kinase receptors (Curtis et al., 1995; Villanueva, 2013). Following binding to their receptors, NTs are internalized along with their receptor and transported via retrograde axonal transport to the cell soma where they initiate multiple survival promoting effects within the nucleus (Curtis et al., 1995; Figure 1).

Evolutionary studies suggest that early members of the NT family evolved approximately 600 million years ago from a common gene, resulting in the sharing of a highly conserved six-cysteine residue domain. Early duplications in the ancestral gene led to the formation of the four members of the NT family (NGF, BDNF, NT-3, and NT4/5). Comparative studies have revealed that while certain members of this NT family have shown extensive divergence (e.g., NGF), others remained largely conserved (e.g., BDNF). BDNF’s high rate of conservation suggests poor evolutionary tolerance for divergence (Götz et al., 1992).

Among NTs, BDNF is the most widely expressed in the brain, affecting neuronal survival, differentiation, axonal path-finding (Reichardt, 2006), regulation of dendritic trafficking to postsynaptic densities (Nakata and Nakamura, 2007), protection against neuronal death in the hippocampus (Pringle et al., 1996), and the induction and maintenance of late phase long-term potentiation (Korte et al., 1995). Treatment of postnatal hippocampal slice cultures with BDNF has been shown to increase spine density in the CA1 area of the hippocampus (Alonso et al., 2004). Moreover, intrahippocampal injection of human BDNF in rats leads to significant improvement in memory in these animals (Alonso et al., 2002). These studies support the notion that BDNF is critically important to the cellular and subcellular mechanisms that underlie learning and memory. Importantly, it has been demonstrated that plasma BDNF is indeed a reliable biomarker for impaired memory in humans (Komulainen et al., 2008).

The synthesis of BDNF occurs primarily in the CNS, initially as a precursor molecule consisting of 250 amino acid residues, a length twice the size of mature BDNF (Götz and Schartl, 1994). This synthesis has been shown to occur in areas with a prominence in cognitive function (e.g., hippocampus, frontal, parietal, and entorhinal cortex areas). Notably, these areas have been shown to synthesize BDNF and its high affinity TrkB receptors or retrogradely transport it to distant regions in the brain. Human gene expression studies have demonstrated that central BDNF synthesis is highest in the cortex and hippocampus, followed by the amygdala, basal forebrain, dorsal vagal complex, hindbrain, and ventral tegmental area of the midbrain (Salehi et al., 2006; Marosi and Mattson, 2014). *TrkB receptor* mRNA expression is equally well distributed and partially follows that of *BDNF*. Moreover, a number of brain regions containing BDNF have the ability to retrogradely transport BDNF from their projection areas. For example, while raphe nuclei in the brainstem of rodents are devoid of significant *BDNF* mRNA, serotonergic 5-HT-ergic neurons in these nuclei can retrogradely transport BDNF to their cell bodies from their projection areas including frontal cortex, occipital cortex, entorhinal cortex, and amygdala (Mufson et al., 1999; Salehi et al., 2007). Similarly, norepinephrinergic (NE-ergic) neurons of locus coeruleus receive retrogradely BDNF from entorhinal cortex and frontal cortex. The histaminergic neurons of supramamillary area can retrogradely receive BDNF from the hippocampal formation and entorhinal cortex.

BDNF is synthesized in the periphery by vascular endothelial cells, T-cells, B cells, monocytes (Kerschensteiner et al., 1999; Nakahashi et al., 2000) and skeletal muscles (Mousavi and Jasmin, 2006). Once released, BDNF can cross the BBB bi-directionally (Pan et al., 1998), resulting in a direct relationship between BDNF levels in plasma and the brain (Karege et al., 2002). While most of the BDNF that is produced peripherally is released into circulation, internalized, and stored in platelets, the BDNF produced by muscles is used locally at the neuromuscular junction (Fujimura et al., 2002; Matthews et al., 2009).

NTs play a vital role in the maintenance of the structural and functional health of neurons that underlie cognition (including those of the hippocampal formation, basal forebrain cholinergic neurons, and NE-ergic neurons of the locus coeruleus). Evidence for this lies in the fact that BDNF is expressed pervasively throughout the brain, readily crosses the BBB, significantly impacts the structure and function of the hippocampal dentate gyrus (DG) via its widely expressed TrkB receptors, and imposes a negative feedback on FNDC5 synthesis (negative feedback). These facts suggest that NTs play a crucial role in cognition and, in conjunction with the evidence that links exercise with cognition, makes it seem plausible that NTs may be significantly involved in the mechanisms that underlie the positive effects of physical activity on cognitive function.

##### Lessons from BDNF polymorphism

**Figure 2:**
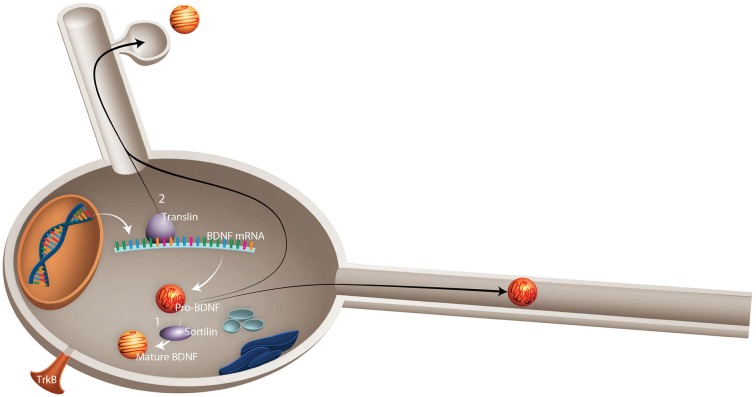
**Schematic representation of possible mechanisms by which val66met substitution in BDNF leads to failed activity-dependent release of BDNF**. Val66met substitution alters the dynamics of interaction between BDNF and two important proteins: **(1)** Sortilin, which is involved in intracellular sorting and pro-neurotrophin signaling. Val66met substitution leads to reduction of BDNF interaction with this protein, which results in mis-sorting into the constitutive secretory pathway instead of activity-dependent release. **(2)** Translin is a highly conserved protein involved in mRNA transport. An exon found in all *BDNF* mRNA splice variants contains a specific translin-binding region, which is essential for appropriate *BDNF* mRNA dendritic targeting. It has been shown that val66met substitution diminishes *BDNF* mRNA interaction with translin, which leads to reduced translocation of *BDNF* mRNA to dendrites (Published from Sanchez et al., 2011).

The most widely studied polymorphism in BDNF is the val66met substitution (Sanchez et al., 2011), a genetic variant that appears to alter susceptibility to neuropathology and response to physical activity. This single nucleotide polymorphism (SNP) results in the substitution of adenine for guanine at position 196 (G196A), resulting in an amino acid substitution of valine to methionine at position 66 of pro-BDNF protein. It seems likely that the val66met substitution alters BDNF processing and, ultimately, BDNF-TrkB signaling. Knowledge of this data has led to the suggestion that the val66met substitution alters the rate of activity dependent BDNF release by affecting proper folding and sorting into secretory vesicles and by reducing intracellular distribution: within the Golgi apparatus, BDNF is sorted into regulated (activity dependent) or constitutive secretory pathways (Sanchez et al., 2011). Sortilin, a member of vacuolar protein sorting 10 family, is highly enriched in the Golgi apparatus and plays an important role in BDNF processing through binding to the pro-domain region of BDNF (Chen et al., 2005). The occurrence of the val66met substitution in this region leads to a significant decrease in BDNF interaction with sortilin (Chen et al., 2005), which contributes to the mis-sorting of BDNF into the constitutive secretory pathway (Figure 2).

In neurons, BDNF is not exclusively synthesized in the somata; rather, fragments of *BDNF* mRNA are generally transported from the cell body to the dendrites for local synthesis (Horch, 2004). Initial *BDNF* gene expression results in the formation of two types of *BDNF* mRNA, those with short 3′ untranslated region (UTR) and others with long 3′ UTR. The long 3′ UTR of the *BDNF* mRNA is transported to the dendrites, whereas the short 3′ UTR remains within the cell body (An et al., 2008). It has been suggested that by reducing interaction with translin, an mRNA binding protein, the G to A substitution in *BDNF* gene leads to failed translocation of *BDNF* mRNA to dendrites (Chiaruttini et al., 2009), reducing its intracellular distribution and contributing to neuropathology. Indeed, the presence of this SNP has been linked to the occurrence of depression in elderly and adolescents, depression in individuals with AD, stroke, anorexia nervosa, anxiety-related disorders, depressive episodes in bipolar disorder, suicidal behavior, schizophrenia, and introversion (Kim et al., 2008; Terracciano et al., 2010). Hippocampal volumetric alterations have also been linked to BDNF polymorphism (Terracciano et al., 2010). Using a meta-analysis of 399 healthy individuals, Hajek et al. (2012) reported a significant reduction in bilateral hippocampal volume in met carriers. In this extensive and longitudinal study in healthy individuals, these authors found val2met substitution predicted the rate of decline in skill task performance and reduction in hippocampal volume (Sanchez et al., 2011). To determine whether the BDNF polymorphism could moderate the effects of physical activity on cognition, Erickson et al. (2008) evaluated the performance of 1,032 midlife volunteers on a battery of tests for memory, learning, and executive processes and physical activity (Erickson et al., 2008). They reported a robust relationship between the BDNF val2met substitution and cognitive performance, with high levels of physical activity ameliorating the deleterious effects of the met allele on working memory performance, suggesting that physical activity can modulate the effects of genetic alterations and provide positive outcomes on cognition. Interestingly, Bryan et al. (2013) demonstrated that the presence of A-alleles in the BDNF polymorphism predicted poor compliance with participation in a high demand physical activity regimen (Bryan et al., 2013). In aggregate, these studies suggest a bidirectional relationship between BDNF polymorphism and physical activity.

**Figure 3:**
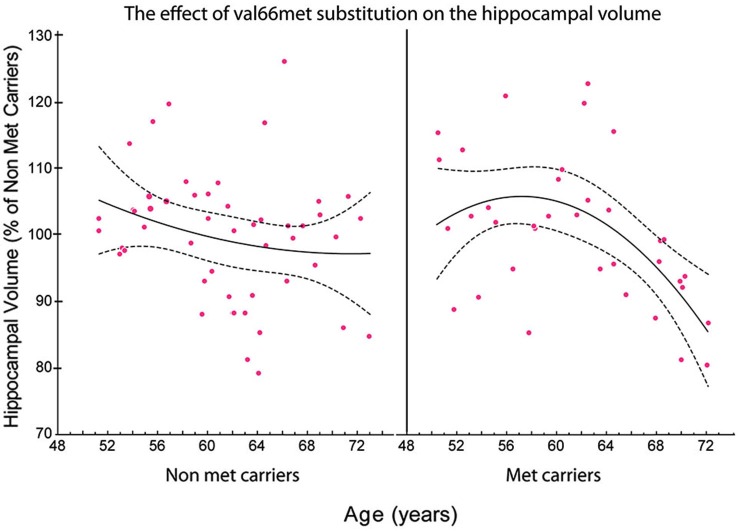
**The relationship between the total volume of the hippocampus and age in met and non-met carriers**. Using a polynomial fitting curve, we found a significant correlation (*r* = −0.447, *P* = 0.0150) between age and the volume of the hippocampus in met carriers. No such correlation was found in non-met carriers (*r* = −0.178, *P* = 0.2639). Furthermore, the slope of the regression in the hippocampal volume of met carriers (slope = −0.038) was twice the value of the slope of regression in non-met carriers (slope = −0.016; Published from Sanchez et al., 2011).

Therefore, the incidence of SNPs should be taken into account when considering the effects of physical activity on BDNF levels and vice versa (Figure 3). Future studies should consider the contribution of BDNF SNPs, particularly rs6265, through well-designed protocols that account for both the population under study and type of exercise administered.

##### BDNF, physical activity, and cognition

Numerous studies in humans and animals have linked the modulation of BDNF with physical activity and cognition. Studies have demonstrated the intensity of exercise training is positively correlated with BDNF plasma levels in young, healthy individuals (Ferris et al., 2007). Resistance exercise has also been shown to elevate serum BDNF levels in young individuals (Yarrow et al., 2010). Moreover, it has been shown that moderate levels of physical activity in people with AD significantly increased plasma levels of BDNF (Coelho et al., 2014). While the source of the exercise-induced-BDNF increases has yet to be fully identified, it appears the increase originates from both central and peripheral sources, with 70–80% of circulating BDNF deriving from the brain and the remaining levels deriving from peripheral sources (e.g., platelets, T-cells, B cells, and monocytes; Rasmussen et al., 2009). In humans, it has been shown that 4 h of rowing activity leads to increased levels of plasma BDNF from the internal jugular (an indicator of central release from the brain) and radial artery (an indicator of peripheral release; Rasmussen et al., 2009). Seifert et al. (2010) reported that basal release of BDNF increases following 3 months endurance training in young and healthy individuals, as measured from the jugular vein. These trends are augmented by rodent studies showing that endurance training leads to increased synthesis of BDNF in the hippocampal formation (Neeper et al., 1995, 1996). Similarly, a significant positive correlation exists for levels of physical activity and *BDNF* mRNA expression in the hippocampus in rats. Exercise induced upregulation of BDNF has been noted in the perirhinal cortex (Hopkins and Bucci, 2010). The levels of BDNF remain upregulated for up to 7 days following endurance training (Berchtold et al., 2005). Widenfalk et al. (1999) have also reported an increase in BDNF-TrkB signaling after treadmill running (Widenfalk et al., 1999). Interestingly, while both physical activity and cognitive training can improve cognitive function, only physical activity can improve BDNF levels in plasma, suggesting that the positive effects of physical activity are mediated by BDNF (Langdon and Corbett, 2012). The question that remains to be addressed is whether blocking the effects of increased BDNF levels block the positive effects of physical activity on cognition. This question has been partly answered by studies showing that blocking the effects of BDNF on TrkB receptors reduced the positive effects of physical activity on cognitive function (Vaynman et al., 2004a,b, 2006).

#### Insulin-like growth factor

Insulin-like growth factor 1 (IGF-1) is an important trophic factor for growth and metabolic reactions. High concentrations of this 70 amino acid polypeptide chain are released by the liver (Clemmons et al., 1995). Epithelial cells comprising the choroid plexus contain IGF receptors, enabling IGF to enter the brain through the CSF pathway (Carro et al., 2000). The main sources of IGF-1 production include muscles, liver, and the brain. Cells with an important role in IGF-1 production in the brain include perivascular macrophages and microglia (Eliakim et al., 1997; Carro et al., 2000). In addition, endothelial cells of the blood vessels and vascular smooth muscle cells have been shown to produce IGF-1. Intracerebroventricular injection of IGF-1 in old rats was able to restore object recognition in these rats (Markowska et al., 1998). IGF-1 levels are low at birth and increase to the age of 20 years, thereafter declining gradually. Peripheral administration of IGF-1 can increase the number of newborn neurons as shown by an increased number of bromodeoxyuridine-positive cells in the DG of rats (Aberg et al., 2000). It appears that the peripheral release of IGF-1 is an important factor in inducing cell proliferation in the DG since chronic infusion of anti-IGF-1 antibody prevented the positive effects of treadmill running in rats (Trejo et al., 2001). Importantly, intra-carotid injection of IGF-1 in rats has been shown to increase *BDNF* gene expression in the hippocampus of these animals (Carro et al., 2000). IFG-1 null mice show a significant reduction in the number of dentate granule cells (DGCs) in the hippocampus (Beck and Hefti, 1995). These mice showed a significant reduction in the number of neurons in CA regions and DGCs in the DG. However, cholinergic neurons in the medial septum and dopaminergic neurons in the striatum remained unchanged (Beck and Hefti, 1995). All mice lacking IGF-1 receptors and a majority of IGF-1 null mice die at birth. Deleting IGF-1 in the liver has made an opportunity to study the effects of peripheral IGF-1. Pappalysin1 is an enzyme that cleaves IGF-1 bound IGF binding protein 4. Deletion of the encoding gene, Pappa, decreases IGF-1 due to an increased abundance of IFFBP4 leading to a significant reduction in body weight and longevity. Multiple large-scale studies in humans have shown that serum levels of IGF-1 are correlated with fitness and as well as body mass indices (Poehlman and Copeland, 1990). Furthermore, animal studies have shown that exercise in rats is associated with increased amounts of IGF-1 in the CSF. IGF-1 acts on muscles by stimulation of amino acid and peripheral glucose uptake helping to maintain skeletal muscle mass.

#### Peroxisome proliferator-activated receptor-gamma coactivator protein-1α

Peroxisome proliferator-activated receptor-gamma coactivator protein-1alpha (PGC-1α) is a transcriptional co-activator of mitochondrial biogenesis and oxidative metabolism in brown fat (Spiegelman, 2007). *Pgc-1α* mRNA has been shown to be highly expressed in the heart, skeletal muscle, and kidney, and to a lesser degree in the liver, pancreas, and brain (Esterbauer et al., 1999). PGC-1α has been shown to induce the activation of uncoupling protein (*UCP1*) gene transcription. This factor is a member of the UCP gene family and is solely expressed in brown adipose and is involved in generating heat (Castillo-Quan, 2012).

*Pgc-1α* null mice develop spongiform neurodegeneration in the striatum and deep layers of the cortex (Ma et al., 2010), and this phenotype has been implicated in PD and Huntington disease (Cui et al., 2006; St-Pierre et al., 2006; Tsunemi and La Spada, 2012), strongly suggesting that PGC-1α plays a significant role in maintaining neuronal function. Due to its role in mitochondrial biogenesis, it has been suggested that it might serve as a neuroprotective agent. Indeed, it has been shown that Pgc-1α knockout animals are more sensitive to 1-methyl-4-phenyl-1,2,3,6-tetrahydropyridine toxicity than controls (St-Pierre et al., 2006).

Since PGC-1α is a transcription factor with no ability to bind to DNA, it has been suggested that it binds to nuclear receptor estrogen-related receptor α (ERRα). This is supported by the fact that physical activity leads to increased ERRα gene expression in the brain (Wende et al., 2005). PGC-1α overexpression leads to increased ERRα gene expression. Disruption of ERRα/PGC-1α leads to reduced levels of *FNDC5* gene expression. Knocking down ERRα blocks PGC-1α induced Fndc5 gene expression (Schreiber et al., 2003).

Recent studies have shown that Pgc-1α is synthesized in muscle cells and induced by exercise and stimulates many of the known markers of exercise in muscles including mitochondrial biogenesis, angiogenesis and fiber-type switching. While chronic long-term treadmill running over 12 weeks leads to increase in Pgc-1α gene expression in muscles, sedentary lifestyle has been shown to be associated with reduced expression of this factor (Handschin and Spiegelman, 2008). Notably, overexpression of Pgc-1α in mice induced similar gene profiling in adipose tissue that is caused by exercise (Boström et al., 2012).

#### Fibronectin type III domain containing 5

**Figure 4:**
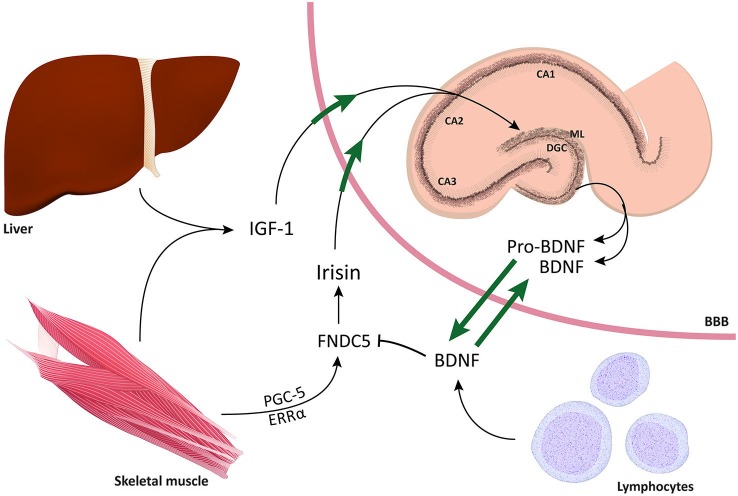
**Schematic representation of mechanisms by which increased physical activity leads to improved cognitive function**. The figure depicts the two compartments alongside the blood brain barrier and the bidirectional relationship of BNDF between central and peripheral compartments. For instance, muscles, liver, and immune cells in the periphery impose a significant influence on the brain, particularly on the DG of the hippocampus. Conversely, BDNF has the ability to easily cross the BBB and influence multiple mechanisms in the periphery. The fact that TrkB receptors have been found in the spinal cord, DRGs, muscles, intestines, and kidneys suggests that BDNF can exert multiple regulatory effects on both sides of the BBB. Through co-activation of PGC1α and ERRα, physical activity induces the production of FNCD5. Following cleavage by a protease, FNDC5 is cleaved into irisin, which has the ability to cross the BBB and induce *BDNF* gene expression in the hippocampus. Notably, it has also been shown that high concentrations of IGF-1 are released by the liver and can, in turn, improve neurogenesis in the DG and induce *BDNF* gene expression.

Fibronectin type III domain containing 5 (FNDC5) is a PGC-1α-dependent myokine that is released during exercise (Boström et al., 2012). FNDC5 is a type 1 transmembrane protein with 3 domains including a transmembrane hydrophobic domain, a 94 aa fibronectin 3 domain, and a signal peptide. Cleavage of FNDC5 by a proteolytic enzyme generates a 12 kDa peptide called irisin (Novelle et al., 2013). Following release, irisin (proteolytic hormone derivative (myokine)) activates oxygen consumption and thermogenesis in fat cells. PGC1α induces the formation of FNDC5 in muscles. For this reason, overexpression of Pgc1α leads to increased production of FNDC5, whereas reduced expression of PGC1α by knocking out the gene leads to reduced production of FNDC5. For example, adenovirus-mediated irisin overexpression can convert white subcutaneous fat into brown fat and, thus, improve energy expenditure (see Figure 4).

In rodents’ brain, *Fndc*5 mRNA has been found primarily in the midbrain, pons, cerebellum, and olfactory bulb. Additionally, very low amounts of *Fndc5* mRNA can be found in the hippocampus. In the periphery, irisin immunoreactivity has been found in skeletal muscles (all types of muscle fibers) and cardiomyocytes. While in the CNS, irisin immunoreactivity has been only found in GABA-ergic purkinje cells in the cerebellum and vestibular nuclei of medulla oblongata, *Fndc5* mRNA has been found extensively in the brain of rodents (Ferrer-Martinez et al., 2002). The fact that FNDC5 immunoreactivity has only been found in the purkinje neurons of rodent brains (Dun et al., 2013) suggests that further investigation into the translation or fate of FNDC5 mRNA is warranted. As for the function of FNDC5, it appears that this molecule is involved in dendritic spine formation (Cheng et al., 2012).

Lecker et al. (2012) found a significant positive correlation between *Fndc5* gene expression in muscles and that of PGC-1α. Furthermore, physical activity led to increase gene expression for FNDC5 in muscles.

Recently, it was shown that exercise induces *Fndc5* gene expression in muscles, and in turn, increases levels of irisin in the circulation. Similarly, exercise leads to increased levels of both FNDC5 and irisin in circulation (Huh et al., 2012; Lecker et al., 2012). Accordingly, it has been shown that endurance exercise leads to increase expression of *Fndc5* in the hippocampus (Wrann et al., 2013).

The exact role of FNDC5 is yet to be fully understood, but it has been shown that it leads to a significant increase in *BDNF* gene expression in cortical cell cultures. IV injection of FNDC5 in mice leads to a significant increase in the *BDNF* gene expression in the hippocampus. Conversely, treating hippocampal neurons with BDNF reduces *FNDC5* gene expression. A positive correlation exists between FNDC5 and *BDNF* gene expression (Wrann et al., 2013). Accordingly, increases in FNDC5 expression in the liver lead to increased levels of *BDNF* mRNA levels in the hippocampus (Wrann et al., 2013). However, direct application of irisin to cultured hippocampal neurons did not increase *BDNF* gene expression suggesting that FNDC5 might have another cleavage product (other than irisin) that can induce *BDNF* gene expression (Xu and Heilshorn, 2013).

## Putative effects of extreme physical activity on the brain and cognitive function

Despite the positive effects of moderate physical activity on the brain, a number of studies have linked extreme exercise to disruption of cellular, metabolic, and hormonal processes and, in turn, to adverse neurological sequelae and cognitive dysfunction. Here, we review cellular mechanisms by which extreme physical activity might interfere with normal neuronal function, particularly those involved in learning and memory.

### Increased reactive oxygen species and cytokine production

The brain comprises approximately 2% of adult human body weight and yet consumes approximately 20% of oxygen at rest (Allaman, 2013). The high rate of oxygen consumption, coupled with the low levels of anti-oxidant enzymes found in the brain, particularly during intense levels of physical activity, increases the vulnerability of the CNS to oxidative stress (Uttara et al., 2009). It has been shown that extreme levels of physical activity generate high levels of reactive oxygen species (ROS), leading to oxidative damage to DNA, RNA, proteins, and lipids (Aguiló et al., 2005). Tsakiris et al. (2006) demonstrated that prolonged forced swimming induced increased levels of ROS in rats (Tsakiris et al., 2006). It has been demonstrated that intense physical activity results in oxidative stress in rodents as shown by a significant increase in brain glutathione peroxidase (Hara et al., 1997) and lipid peroxidation (Somani and Husain, 1996). Moreover, Rosa et al. (2007) demonstrated young adult mice undergoing 10 days of intense and exhaustive running program exhibited a high index of brain oxidative stress and impaired memory as assessed by fear conditioning. Recapitulating these effects in humans, Aguiló et al. (2005) demonstrated that intense mountain exercise leads to the generation of oxidative stress and high levels of ROS. While vitamin E, an essential anti-oxidant, typically inhibits the production of ROS during lipid peroxidation, intense physical activity in humans leads to a significant increase in vitamin E turnover that weakens antioxidant defence (Mastaloudis et al., 2001). Thus, although moderate levels of physical activity can enhance the antioxidant defence mechanisms, extreme levels of physical activity can deplete anti-oxidant reserve (Mastaloudis et al., 2001), leading to adverse neurological effects (Gomez-Cabrera et al., 2008). Alternatively, cytokines may modulate the negative effects of extreme physical activity. IL-6 is directly released by muscles during physical activity and activates the release of adrenocorticotropic hormone (ACTH) from the pituitary gland and increases cortisol levels (Mastorakos et al., 2005). Together, these studies suggest that extreme levels of physical activities may weaken the immune system either by reducing anti-oxidant defence or by altering cortisol levels.

### Increased corticosteroid signaling

Cortisol is a glucocorticoid (GC) that is released from the adrenal gland in response to stress (Kudielka et al., 2009). The release of cortisol from the adrenal gland is regulated by the release of ACTH from the pituitary gland. Under physiological conditions, cortisol, in conjunction with epinephrine and norepinephrine, prepares an individual for the “fight or flight response”, enabling rapid shifting of blood flow toward large skeletal muscles and permitting an individual to flee threatening situations. Cortisol also plays a significant role in memory processes (Tollenaar et al., 2009), with areas of the brain important for memory (e.g., hippocampus, prefrontal cortex, amygdala) expressing high levels of GC receptors (Ramos and Arnsten, 2007). In the hippocampal region, excessive GCs levels have been linked to a significant reduction in neurogenesis (Liu et al., 2003), suppression of LTP in excitatory synapses (Setiawan et al., 2007), cell death through apoptosis (Zhao et al., 2007), and extensive dendritic re-organization in the prefrontal cortex (Cook and Wellman, 2004).

Historically, it has been assumed that physical activity lowers GC levels and attenuates the adverse effects of stress (Cornil et al., 1965); however, more recent evidence suggests the positive effects of physical activity peak at upper-middle intensity levels (Soya et al., 2011), with intense levels of physical activity being associated with adverse effects (Tomporowski, 2003; Taverniers et al., 2010, 2013). Indeed, it has been shown that the magnitude of cortisol response is both positively and linearly related to the intensity and duration of physical activity (Jacks et al., 2002; Tauler et al., 2014). Similarly, elevated levels of ACTH have been detected in the circulation after extreme exercise (plasma adrenocorticotropin and cortisol responses to submaximal and exhaustive exercise). It has been showed that both extreme physical activity paired with psychological stress is linked to concomitant increases in cortisol and impaired memory (Tomporowski, 2003; Taverniers et al., 2010, 2013).

## Lessons for intervention

While the interactions between mind, brain, and body have been conjectured for centuries, only recently have we begun to understand the putative molecular mechanisms of such a relationship. Ratification of the positive benefits of physical activity are now evident in studies demonstrating that increased physical activity can significantly improve longevity (Bronnum-Hansen et al., 2007), decrease the incidence of multiple chronic disorders (Mokdad et al., 2004; Goodwin, 2008; Arida et al., 2009) and delay age-related degeneration of brain tissue (Colcombe et al., 2003). These benefits appear to be enhanced during times of high cognitive demand, stress, and illness.

Also drawing increased attention in recent years is the positive role that physical activity plays in increasing learning and memory (van Praag et al., 1999). A meta-analysis of 18 longitudinal studies has revealed that physical activity improved overall cognitive function in humans (Vaynman et al., 2006). Moreover, it has been demonstrated that physical activity can ameliorate the effect of neurological diseases characterized by cognitive deficits, particularly in AD, PD, and psychiatric illnesses such as depression and schizophrenia (Rolland et al., 2007). For example, *BDNF* gene expression levels are commonly reduced in the normal aging population and in people with AD (Phillips et al., 1991); yet increased levels of physical activity appear to restore BDNF to basal levels and, in turn, improve cognitive function in the aging (Laurin et al., 2001) and AD patients (Lautenschlager et al., 2008).

In the CNS, exercise has been shown to increase adult neurogenesis in the DG of the hippocampus, improve dendritic complexity and synaptic plasticity in the perforant path that carries information from the entorhinal cortex to the DG (Eadie et al., 2005), and increase angiogenesis. All these effects can be achieved through the improved release and signaling of neurotrophins, particularly BDNF. Scharfman et al. (2005) have demonstrated that BDNF administration into the DG increases angiogenesis. Similarly Tg2576 mouse models of AD show increased levels of IL-1β and TNF in the hippocampus compared with controls. IL-1β was found to be elevated in AD patients compared with controls (Alvarez et al., 1996). Notably, this cytokine can increase *APP* gene expression and protein (Griffin et al., 2006). Three weeks of physical activity improved cognitive function as assessed in Morris Water maze in mice, and reduced the brain expression of pro-appoptic factors like caspase-9, caspase-3 and Bax and increased the expression of anti-apoptotic factors like Bcl-2 in these mice.

At the interface of physical activity and enhanced cognitive function are NTs, particularly BDNF. BDNF is upregulated following physical activity, with approximately 70–80% of the additional expression derived centrally. Notably, activity-induced upregulation of BDNF has been found primarily in the hippocampus, a brain region critically important for learning and memory.

The questions that have been raised are on the nature of specificity and on the increase in *BDNF* gene expression in the hippocampus. A number of studies have indicated that physical activity causes a significant increase in *BDNF* gene expression most robustly in the hippocampus (Cotman et al., 2007). This may suggest that hippocampal neurons particularly DGCs have receptors that would bind to a yet to be identified factor that is released in the periphery to connect physical activity at the periphery to gene expression as well as function in the CNS, particularly the hippocampus. The fact that FNDC5 injected systemically causes increased *BDNF* expression in the hippocampus while treating cultured hippocampal neurons with this compound does not induce *BDNF* gene expression in these cells support this notion (Wrann et al., 2013). The DG region of the hippocampus is one of the most vulnerable regions to aging and neurodegeneration. However, this region is one of the few regions that retain neurogenesis capabilities in adulthood, a process that is significantly enhanced by exercise.

As we showed here (Sanchez et al., 2011), val2met polymorphism in the pro-domain of BDNF predicts the rate of failure in skill task performance and the volume of the hippocampus in healthy individuals. Multiple studies have shown that met carriers show a significant reduction in the amount of BDNF released during activity. Both BDNF and IGF-1 play a significant role in cognition and motor function in humans. For these reasons, the effects of genetic variations on physical activity must be taken into consideration in any clinical trial, as polymorphisms modulate the individualized response to physical activity. Questions regarding the role of genetic influences on the association between response to physical activity and cognitive function remain. Testing for genetic and environmental factors may be important because cognitive decline appears to be highly heritable and physical activity levels seem to be influenced by both factors (e.g., socioeconomic status, nutrition, demographic).

Moreover, a better understanding of the relationship between genetic profiles and distinct response to illness will permit health care professionals to prescribe individualized exercise regimens according to genetic profile. Such would enable the diagnosis and treatment of an individual’s personal response to aging and illness (Booth and Laye, 2009). For example, it has been shown that val66met substitution reduces the excitability of primary motor cortex following a repetitive motor task compared with val66val individuals (Kleim et al., 2006). People with depression show diminished levels of BDNF in their serum (Huang et al., 2008). It has been shown that the use of repetitive transcranial magnetic stimulation (rTMS) in depression can be linked to increased BDNF levels (Zanardini et al., 2006), and yet met carriers appear to be significantly less responsive to this procedure (Bocchio-Chiavetto et al., 2008). Expanded use of rTMS in the clinical setting and the potential ability of this technology to induce *BDNF* expression suggest a means to improve BDNF levels in individuals whose physical limitations avert participation in physical activity.

The identification of irisin suggests new avenues for pharmacological intervention. It has been shown that IV administration of irisin increases BDNF in mice, a potential treatment for improved BDNF levels in those individuals who because of physical impairments cannot participate in physical activity.

Plasma BDNF measures are highly variable between individuals. Thus, meaningful studies must account for age, gender, ethnicity, body weight (Komori et al., 2006), and activity level.

A key area of future research will be to refine cognitive studies so as to investigate the genetically determined personalized response to physical activity to increase relevance and translatability to humans. Uncovering the molecular and cellular linkages between physical activity and cognition is critical to advancements in studies of aging and neurodegeneration. A better understanding of these mechanisms will enable the development of pharmaceuticals, particularly for those who are activity limited (coma, spinal cord injury, etc.). Moreover, a better understanding will permit healthcare professionals to provide individualized prescriptions for patients after considering their genetic and proteomic profiles, both of which determine an individual’s personal response to aging and illness (Booth and Laye, 2009).

## Conclusions

There is an urgent need to develop pharmacological and non-pharmacological methods to improve the status of neurons and their dendritic terminals given the increase in individuals affected by neurodegenerative disorders. Physical activity offers an affordable and effective method to improve cognitive function in all ages, particularly the elderly who are most vulnerable to neurodegenerative disorders. The dual effects of chronic and acute physical activity on inflammatory processes, particularly in those individuals with an underlying inflammatory condition, must be better understood such that the nature of physical activity and its inducing health benefits can be harnessed for therapeutic purposes in vulnerable populations.

Further refinement of the mechanisms by which myokines are released by peripheral muscles during exercise could improve our understanding of mechanisms of BDNF synthesis by the DG and could potentially lead to the identification of new and therapeutically-important factors that mediate these effects. Moreover, since the majority of BDNF synthesis occurs in the hippocampus, there might be new technologies developed in the future to quantify the release of BDNF from the brain and not in whole circulation. The availability of novel nanotechnological methods to collect blood samples locally at the cellular level would refine further knowledge about the type and intensity of physical activities that induce BDNF synthesis in the brain (Song et al., 2012). Expansion of the use of new and high throughput DNA sequencing systems would facilitate better the study of the effects of different polymorphisms, particularly val2met in the *BDNF* gene. Having access to information regarding one’s genetic profile and individualized response to exercise would constitute the first steps toward achieving our primary goal of delivering personalized medicine.

## Conflict of interest statement

The authors declare that the research was conducted in the absence of any commercial or financial relationships that could be construed as a potential conflict of interest.

## Abbreviations

AD: Alzheimer’s disease
BBB: blood brain barrier
BDNF: brain-derived neurotrophic factor
CRP: C-reactive protein
CNS: central nervous system
CSF: cerebrospinal fluid
CXCL8: chemokine (CXC Motif) ligand 8
XCL12: chemokine (CXC Motif) ligand 12
CXCL8R1: CXC chemokine receptor 1
CXCL8R2: CXC chemokine receptor 2
DG: dentate gyrus
DGCs: dentate granule cells
ERRα: estrogen-related receptor α
FNDC5: fibronectin type III domain containing 5
GCs: glucocorticoids
IGF-1: Insulin-like growth factor 1
IL-6: interleukin 6
IL-8: interleukin 8
NGF: nerve growth factor
NT-3: neurotrophin-3
NT4/5: neurotrophin-4/5
NTs: neurotrophins
NE-ergic: peroxisome proliferator-activated receptor-gamma coactivator protein-1alpha
NTs: nor-epinephrinergic
PD: Parkinson’s disease
PGC-1α: peroxisome proliferator-activated receptor-gamma coactivator protein-1alpha
rTMS: repetitive transcranial magnetic stimulation
ROS: reactive oxygen species
SNP: single nucleotide polymorphism
Trk: tropomyosin related kinase
TrkB: tropomyosin related kinase B belonging to receptor tyrosine kinase family
TNF: tumor necrosis factor
UCP: uncoupling protein
UTR: untranslated region

## Notes

*Received: 22 April 2014; accepted: 02 June 2014; published online: 17 June 2014*.

Copyright © 2014 Phillips, Baktir, Srivatsan and Salehi. This is an open-access article distributed under the terms of the Creative Commons Attribution License (CC BY). The use, distribution or reproduction in other forums is permitted, provided the original author(s) or licensor are credited and that the original publication in this journal is cited, in accordance with accepted academic practice. No use, distribution or reproduction is permitted which does not comply with these terms.
